# Most of moderate to severe ulcerative colitis patients in academic clinical practice still do not qualify for randomized controlled trials

**DOI:** 10.1093/crocol/otag092

**Published:** 2026-08-04

**Authors:** Sandra Elmasry, Shabana Pasha, Manreet Kaur, Jonathan A Leighton, Suryakanth R Gurudu, Christina Ha

**Affiliations:** Department of Medicine, Mayo Clinic Arizona , 13400 East Shea Boulevard Scottsdale, AZ 85259, United States; Division of Gastroenterology and Hepatology, Mayo Clinic Arizona, 13400 East Shea Boulevard Scottsdale AZ 85259, United States; Division of Gastroenterology and Hepatology, Mayo Clinic Arizona, 13400 East Shea Boulevard Scottsdale AZ 85259, United States; Division of Gastroenterology and Hepatology, Mayo Clinic Arizona, 13400 East Shea Boulevard Scottsdale AZ 85259, United States; Division of Gastroenterology and Hepatology, Mayo Clinic Arizona, 13400 East Shea Boulevard Scottsdale AZ 85259, United States; Division of Gastroenterology and Hepatology, Mayo Clinic Arizona, 13400 East Shea Boulevard Scottsdale AZ 85259, United States

**Keywords:** ulcerative colitis, inflammatory bowel disease, randomized clinical trials, Janus kinase inhibitors, sphingosine-1 phosphate receptor agonists, American Gastroenterological Association

## Abstract

**Background:**

While biologic and small molecule therapies have expanded treatment options for moderate to severe ulcerative colitis (UC), prior research revealed that only 26% of patients in routine practice would have qualified for pivotal randomized controlled trials (RCTs) due to stringent eligibility criteria. This study aimed to evaluate contemporary clinical trial eligibility for UC patients in a tertiary care setting based on more recently approved therapies.

**Methods:**

We performed a retrospective cohort study of adult patients with moderate to severe UC (total Mayo score ≥6) seen between January 2022 and February 2023. Inclusion and exclusion criteria from seven phase III RCTs conducted between 2012 and 2022 (eg, VARSITY, UNIFI, OCTAVE, LUCENT) were applied to determine patient eligibility and identify reasons for ineligibility.

**Results:**

Of 150 patients (mean age 44.9 years; 55.7% male), only 42.7% were eligible for at least one RCT. The most common exclusion factor was current or prior medication use, followed by anemia, dysplasia, and disease extent. Trial eligibility did not significantly differ by age, gender, or disease duration.

**Conclusions:**

Despite modest improvements since 2012, over half of real-world UC patients remain ineligible for current RCTs. Expanding inclusion criteria, especially regarding prior medication exposure, and adopting more inclusive trial designs may enhance external validity. Addressing disparities in trial access and participation remains essential to ensuring equitable representation and improving clinical applicability of RCT findings.

## Graphical Abstract

**Graphical Abstract:**
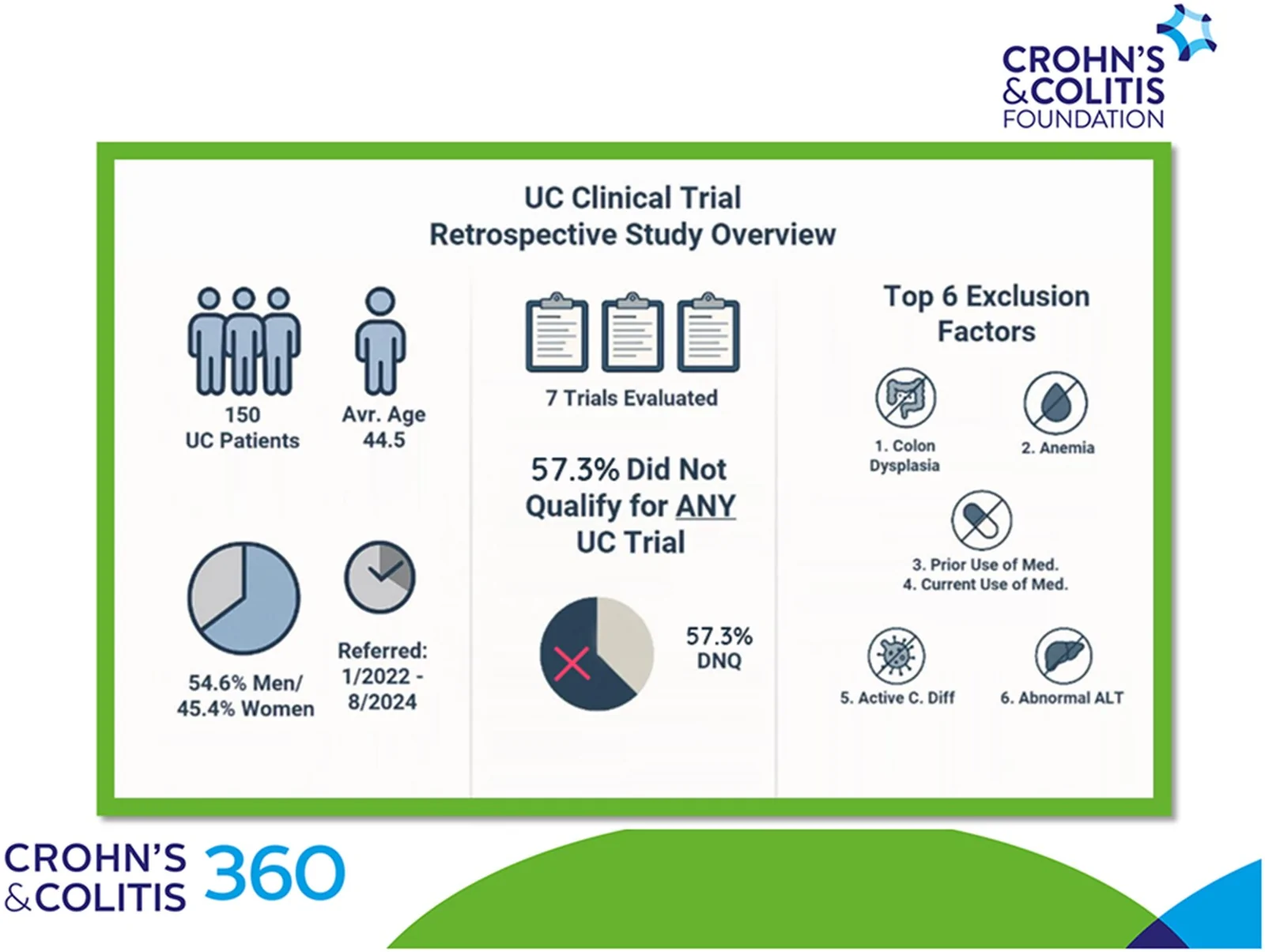

## Introduction

Ulcerative colitis (UC) is a chronic immune-mediated inflammatory bowel disease with the potential for significant morbidity and impact on patients’ quality of life. The goals of UC treatment are to induce and then maintain remission, ideally with mucosal healing and prevention of disability.^1^ In the past twenty years, biologic therapies including TNF-alpha antagonists, anti-integrin and anti-interleukin therapies have demonstrated efficacy through randomized clinical trials (RCT) for patients with moderate to severe UC. However, it was previously noted that in an outpatient practice only 26% of patients with moderate to severe UC would qualify for pivotal RCTs from 2002-2012 era which raised concern regarding the external validity of RCTs due to stringent inclusion and exclusion criteria necessary to have greater internal validity of results.^2^ However, since 2012 additional advanced therapies for UC have been approved for moderate to severe UC patients including Janus kinase inhibitors (JAK), anti-interleukin 23 agents, and sphingosine-1 phosphate receptor agonists (S1P). This study’s aim was to provide an updated assessment of UC clinical trial eligibility based on inclusion and exclusion criteria of the clinical trials for these newer drugs with alternate mechanisms of action.

## Methods

We performed a retrospective cohort study of adult patients with moderate to severe UC presenting to a tertiary IBD referral center for diagnosis or adjustment of medical therapy from January 2022 to August 2024. Patients were included if they had a total Mayo score of ≥6 and a Mayo endoscopic subscore of 2 (moderate) or 3 (severe), with a disease duration of at least 3 months, which was a major inclusion criterion across all trials. Seven published phase III RCTs for moderate to severe UC from 2012 to 2022 were included in this analysis: TRUE NORTH, U-ACCOMPLISH & U-ACHIEVE,^3^ OCTAVE,^4^ ELEVATE,^5^ UNIFI,^6^ VARSITY,^7^ and LUCENT.^8^ A summary of each trial, including the medication studied, design, and trial years is provided in Table S2. Inclusion and exclusion criteria from these trials were identified and represented in Table S3. This was systematically applied to our study population to assess overall patient eligibility. Patients were evaluated for eligibility at the time of referral based on available recent laboratory results, endoscopic findings, and symptom assessment. For patients who did not meet eligibility criteria, the most common exclusion variables were identified.

## Results

A hundred and fifty patients with moderate to severe UC (defined as a total Mayo score ≥ 6) presenting for initial or change in medical treatment during the 32-month period were identified. Patients average age of 44.9 ± 18.5, 55.7% Men/44.3% Women with an average disease duration 11.6 yrs. ± 11.1 yrs. Disease extent of our patient population was: proctitis, 8.9%; left sided, 20.3%; and extensive disease, 70.8% (Table S1). There was no difference in age, gender, disease duration or extent between those who would qualify and those who would not qualify for an RCT. Overall, only 42.7% (*n* = 64) of patients would have qualified for at least one RCT. Among those, 11.3% for two trials, and another 11.3% for three or more trials.

**Figure 1:**
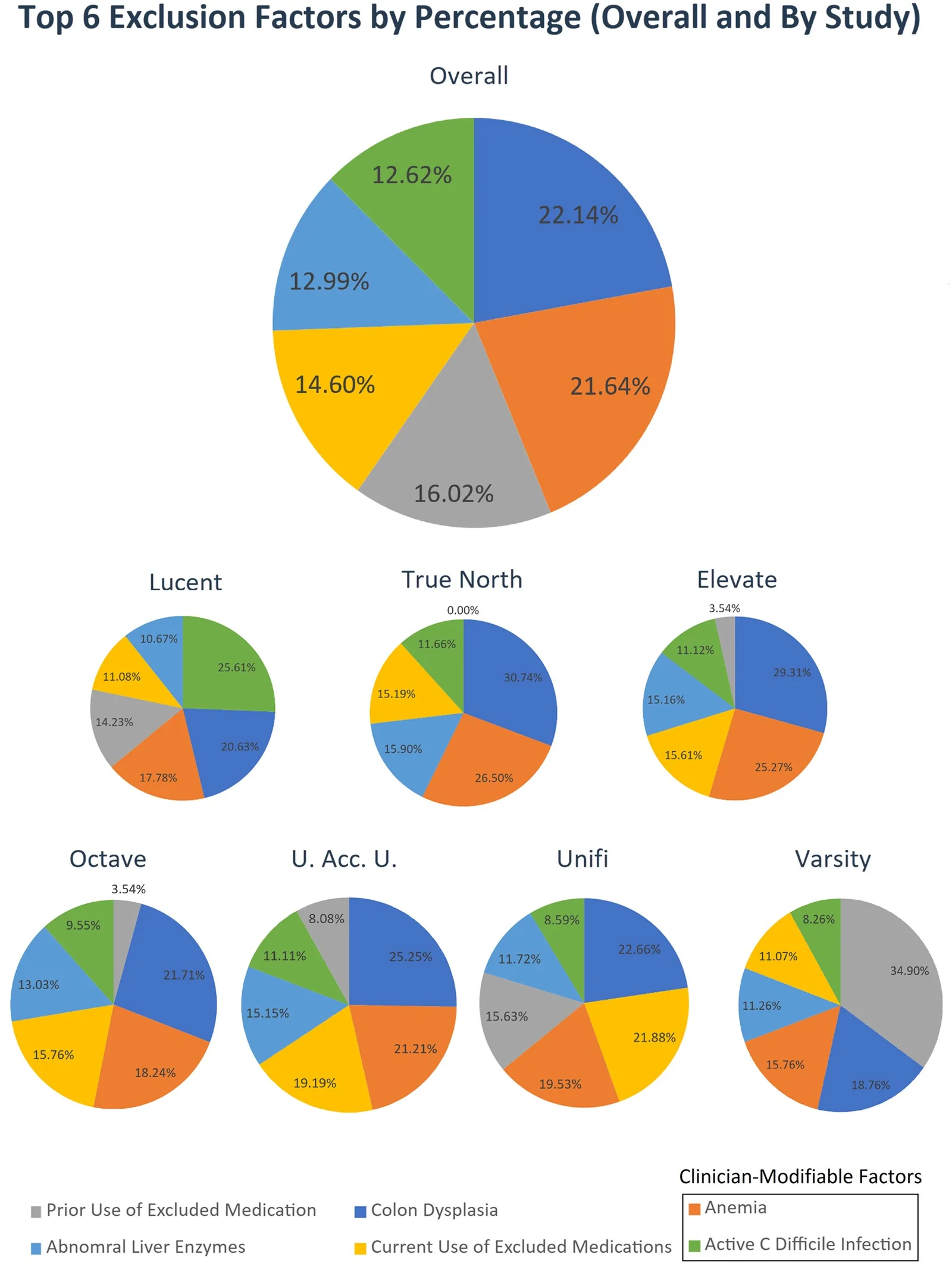
Top exclusion factors by each study and overall.

Among the study population, the OCTAVE trial had the largest percentage of patients who would qualify (26.7%) while the LUCENT and ELEVATE trial had the least percentage of patients that would qualify (11.3%) (Table 1). The most common reason for trial ineligibility was current use or recent exposure to a medication as specified by the trial. Other more common exclusion factors included a history of colonic mucosal dysplasia or cancer excluded per trial definition, degree of anemia, prior medication exposure, active/recent infection, and disease extent. Figure 1 summarizes the distribution of these six exclusion factors overall and by individual trial. Percentages reflect the relative contribution of each factor among these six exclusion criteria, not the overall population or all exclusion reasons.

**Table 1:** Percentage of patients that qualify or do not qualify in the UC Clinical trials from the last 10 years.

***Table 1A: Trial eligibility for uc patients based on published inclusion/exclusion criteria***		
	Qualify	Would not qualify
**ANY Phase III UC RCT**	64 (42.7%)	86 (57.3%)
** VARSITY (adalimumab vs vedolizumab)**	19 (12.7%)	131 (87.3%)
** UNIFI (ustekinumab)**	33 (22.0%)	117 (78.0%)
** U-Accomplish & U-Achieve (upadacitinib)**	24 (16.0%)	126 (84.0%)
** OCTAVE (tofacitinib)**	40 (26.7%)	110 (73.3%)
** ELEVATE (etrasimod)**	17 (11.3%)	133 (88.7%)
** TRUE NORTH (ozanimod)**	29 (19.3%)	121 (80.7%)
** LUCENT (mirikizumab)**	17 (11.3%)	133 (88.7%)

## Discussion

The introduction of more advanced therapies over the last decade has been essential to improving patient outcomes by providing more options for patients. RCTs are essential to demonstrate effectiveness and safety prior to FDA approval and these results are foundational for developing guidelines for clinical practice and shared decision-making with patients. Compared to results from 2012, our study findings demonstrate potential improvement with 42.7% of patients now considered trial eligible for at least one study although there remains considerable potential for improvement to include a broader range of patients.

Our study’s findings emphasize that the exclusion of patients from randomized clinical trials (RCTs) is primarily driven by their current or historical use of medications. However, other factors such as Clostridium difficile infection, colon dysplasia, anemia, and the extent of the disease also play pivotal roles, highlighting the complex landscape of patient eligibility. These criteria can be categorized as either modifiable or non-modifiable.

Non-modifiable exclusion criteria are factors inherent to the patient or disease—such as colon dysplasia or disease extent—that cannot be altered by clinicians or addressed through changes in study design. In contrast, modifiable exclusion criteria represent factors for which intervention may improve trial eligibility and can be further categorized based on whether modification occurs at the clinician or trial-design level.

Clinician-modifiable factors, such as anemia or infection, can often be identified early and addressed through supportive care measures (eg, iron supplementation or antimicrobial therapy), allowing patients to later qualify for trial participation. In our analysis, clinician-modifiable factors accounted for 34.26% of the six most common exclusion criteria. These factors are therefore important to recognize, as they may lead to temporary delays in treatment initiation or trial enrollment rather than permanent exclusion.

Trial-design-related factors may also be further conceptualized as modifiable or non-modifiable. However, within this category, some exclusions remain effectively non-modifiable (eg, exceeding the allowable number of prior biologics or prior exposure to the investigational agent), whereas others—such as current medication use—may be potentially modifiable through defined washout periods. Importantly, washout requirements must be carefully considered, as interrupting therapy in patients with moderate to severe UC may increase the risk of disease flare or progression. Taken together, these findings highlight that expanding trial eligibility will likely require both optimization of clinician-modifiable factors and thoughtful reconsideration of trial design–related restrictions to meaningfully improve patient participation.

Randomized controlled trials (RCTs) face the challenging task of maintaining internal validity to insure accurate and reliable results, often at the expense of external validity, thereby limiting the generalizability of their findings to broader patient populations. Additionally, referral center bias from tertiary referral center certainly impacts trial eligibility as the patient population tends to skew toward more refractory, more medication-experienced with a greater likelihood of disease or medication-related complications. Demographic factors such as ethnicity, educational level, language comprehension, occupation, insurance status, and geography are known to impact successful clinical trial recruitment. Additionally, a sizeable number of potentially eligible patients lack access to an established IBD clinical trials program as most IBD RCTs take place in a small number of clinical trial programs, often located within metropolitan areas.^3^

Concerns regarding the accessibility of trials and the underrepresentation of minorities in RCTs were the focus of the American Gastroenterological Association (AGA) IBD roundtable with discussions emphasizing the need to expand RCT networks and provide support to overcome healthcare disparities and socioeconomic differences.^9^ Other trial-modifiable factors that should be explored include shorter washout phases, innovative and more inclusive trial designs, especially during the maintenance phases which account for the prior medication exposures expected in a moderate to severe UC patient population. Additionally, there must be discussions about eliminating placebo arms which lead to ethical dilemmas as placebo-treated patients are more likely to experience disease flares with associated adverse events particularly when existing FDA approved agents could have been given instead.^10^ Alternative randomized controlled trial designs, such as open-label trials, should be considered to ensure all patients receive active treatment.

Like many tertiary referral centers, our institution is not immune to referral bias and cares for patients with more complex, treatment-refractory disease and greater prior therapy exposure. As a result, the rate of trial ineligibility in our cohort may be higher than in community practice, and true eligibility in the broader IBD population may be underestimated. Therefore, the generalizability of our findings to community-based settings should be interpreted with caution.

Over the past decade, there has been some improvement in patient inclusion within randomized clinical trials, yet significant challenges remain. Expanding trial networks and addressing healthcare disparities are crucial steps to enhance patient participation and external validity. Future efforts must prioritize inclusive and adaptive trial designs to better represent the diverse patient population.

## Supplementary Material

otag092_Supplementary_Data

## Data Availability

Analysis was compiled from Mayo Clinic’s EMR (Epic) reviewing adult patients with moderate to severe UC presenting to a tertiary IBD referral center for diagnosis or adjustment of medical therapy from January 2022 to August 2024. Data is not publicly available.
