# Supplementary material for: Most of moderate to severe ulcerative colitis patients in academic clinical practice still do not qualify for randomized controlled trials
Source: Crohns Colitis 360. 2026 Aug 4;8(3):otag092. doi: 10.1093/crocol/otag092 (PMC13499232; doi:10.1093/crocol/otag092)
Supplement: Supplementary file 1 [file otag092_supplementary_data.zip › Supplemental Tables.docx]

|  | | Qualify | Would Not Qualify | Both |
| --- | --- | --- | --- | --- |
|  | | (n = 64) | (n = 86) | (n = 150) |
| Age, *y* (mean ± SD) | | 45.8 ± 17.5 | 44.3 ± 19.7 | 45.0 ± 18.8 |
| Gender | | 32 M/32 F | 50 M/36 F | 82 M/68 F |
| Disease Duration (*y*) | | 11 (range, 1-41) | 11 (range, 1-55) | 11 (range, 1-55) |
| Disease Extent | |  |  |  |
|  | Proctitis | 1 (1.6%) | 2 (2.3%) | 3 (2.0%) |
|  | Left-Sided | 17 (26.6%) | 24 (27.9%) | 41 (27.3%) |
|  | Extensive | 46 (71.9%) | 60 (69.8%) | 106 (70.7%) |

Supplemental Table 1: Patient characteristics including age, gender and disease extent

| **Trial Name** | **Enrollment period** | **Medication Tested** | **Study Design** |
| --- | --- | --- | --- |
| VARSITY | Sep2015-Sep 2017 | Vedolizumab vs Adalimumab | Phase 3, randomized, double-blind, double-dummy, active-controlled |
| UNIFI | May 2015- Sept 2016 | Ustekinumab | Phase 3, randomized, double-blind, placebo-controlled |
| U-ACCOMPLISH &U-ACHIEVE | Oct 2018-Sept 2020 | Upadacitinib | Phase 3, randomized, double-blind, placebo-controlled |
| OCTAVE | Nov 2012-Dec 2014 | Tofacitinib | Phase 3, randomized, double-blind, placebo-controlled |
| ELEVATE | Sept 2018-Sept 2021 | Etrasimod | Phase 3, randomized, double-blind, placebo-controlled |
| True North | Dec 2015-Oct 2019 | Ozanimod | Phase 3, randomized, double-blind, placebo-controlled |
| Lucent | Feb 2019-Apr 2021 | Mirikizumab | Phase 3, randomized, double-blind, placebo-controlled |

Supplemental Table 2: Summary of all RCT trails from 2012-2022.

| Study | Exclusion Criteria | Analysis Group |
| --- | --- | --- |
| **Elevate** | Patient cannot have an abnormal AST/ ALT (>2x ULN). | Abnomral Liver enzymes |
|  | Patient cannot have an active or recent (within 30 days) C difficile infection. | Active C diff Infection |
|  | Patients must be less than 80 years in age. | Age |
|  | Patient cannot have anemia (Hb < 9). | Anemia |
|  | Patient cannot have cardiovascular disease (MACE, stroke, CHF, arrythmia). | Cardiovascular Disease |
|  | Patient cannot be chronically taking NSAIDs. | chronic NSAIDS use |
|  | Patient cannot have history of colon dysplasia or cancer. | Colon Dysplasia |
|  | Patient cannot have history of colon dysplasia or cancer. | Colon Resection |
|  | Patient cannot be currently taking more than 20mg of steroids. | Current Use of Excluded Medications |
|  | Patient cannot be starting or be on a not stable dose of Thiopurine/ MTX. | Current Use of Excluded Medications |
|  | Patient cannot be currently taking aTNF. | Current Use of Excluded Medications |
|  | Patient cannot be currently taking VDZ. | Current Use of Excluded Medications |
|  | Patient cannot be currently taking UST/ RISA. | Current Use of Excluded Medications |
|  | Patient cannot be currently taking a Jak inhibitor (tofa, upa). | Current Use of Excluded Medications |
|  | Patient cannot be taking topical/ rectal steroids (budesonida foam, hydrocort enema). | Current Use of Excluded Medications |
|  | Patient cannot be taking topical/ rectal 5-ASA (Canasa, Rowasa). | Current Use of Excluded Medications |
|  | Patient cannot have diabetes 1 or poorly controlled diabetes 2 (A1c elevated, end organ complications) or macular edema. | Diabetes 1 or Poorly Controlled 2 |
|  | Patient cannot have disease extent limited to proctitis (involvement up to 15 cm). | Disease Extent |
|  | Patient cannot have history of thromboembolic event (arterial or venous). | History Of Thromboembolic Event |
|  | Patient cannot have history of rheumatologic disease (including lupus). | History of Rheumatologic Disease |
|  | Patient cannot have history of infection (zoster, hepatitis, HIV, TB, or serious infection). | Infection History |
|  | Patient cannot have Leukopenia (WBC < 3.5). | Leukopenia |
|  | Patient cannot have malignancy history (solid or liquid). | Malignancy History |
|  | Patient cannot be taking non-IBD immunosuppression medication including transplant medication (e.g. anakinra, abetacept, tacrolimus, cyclosporine, mycophenolate) . | Non-Ibd Immunosuppression Incl Txplt Meds |
|  | Patient cannot have Nontuberculuous mycobacterial infection or serious opportunistic infection. | Nontuberculous Mycobacterial Infection or Serious Opportunistic Infection |
|  | Patient cannot be currently taking or have taken an S1P. | Prior Use of Excluded Medication |
|  | Patient cannot be currently taking or have taken natalzumab. | Prior Use of Excluded Medication |
|  | Patient cannot have renal insufficiency (GFR > 30). | Renal Insuffiency |
| **Lucent** | Patient cannot have an abnormal AST/ ALT (>2x ULN). | Abnomral Liver Enzymes |
|  | Patient cannot have an active C Difficile Infection. | Active C diff Infection |
|  | Patients must be less than 80 years in age. | Age |
|  | Patient cannot have anemia (Hb < 9). | Anemia |
|  | Patient cannot be chronically taking NSAIDs. | chronic NSAIDS use |
|  | Patient cannot have history of colon dysplasia or cancer. | Colon Dysplasia |
|  | Patient cannot have had a colon resection or ostomy. | Colon Resection |
|  | Patient cannot be taking more than 30 mg of prednisone. | Current Use of Excluded Medications |
|  | Patient cannot be currently taking aTNF. | Current Use of Excluded Medications |
|  | Patient cannot be currently taking VDZ. | Current Use of Excluded Medications |
|  | Patient cannot be taking non-IBD immunosuppression medication including transplant medication (e.g. anakinra, abetacept, tacrolimus, cyclosporine, mycophenolate) . | Current Use of Excluded Medications |
|  | Patient cannot be taking topical/ rectal steroids (budesonida foam, hydrocort enema). | Current Use of Excluded Medications |
|  | Patient cannot be taking topical/ rectal 5-ASA (Canasa, Rowasa). | Current Use of Excluded Medications |
|  | Patient cannot have diabetes 1 or poorly controlled diabetes 2 (A1c elevated, end organ complications) or macular edema. | Diabetes 1 or Poorly Controlled T2DM |
|  | Patient cannot have disease extent limited to proctitis (involvement up to 15 cm). | Disease Extent |
|  | Patient cannot have history of thromboembolic event (arterial or venous). | History Of Thromboembolic Event |
|  | Patient cannot have history of rheumatologic disease (including lupus). | History of Rheumatologic Disease |
|  | Patient cannot have history of infection (zoster, hepatitis, HIV, TB, or serious infection). | Infection History |
|  | Patient cannot have Leukopenia (WBC < 3.5). | Leukopenia |
|  | Patient cannot have malignancy history (solid or liquid). | Malignancy History |
|  | Patient cannot have Nontuberculuous mycobacterial infection or serious opportunistic infection. | Nontuberculous Mycobacterial Infection or Serious Opportunistic Infection |
|  | Patient cannot be taking or have taken UST/ RISA. | Prior Use of Excluded Medication |
|  | Patient cannot have renal insufficiency (GFR > 30). | Renal Insuffiency |
|  | Patient cannot be starting or be on a not stable dose of Thiopurine/ MTX. | Thiopurine/ MTX Use |
|  | Patient cannot have cardiovascular disease (MACE, stroke, CHF, arrythmia). | Uncontrolled Cardiovascular Disease |
| **Octave** | Patient cannot have an abnormal AST/ ALT (>2x ULN). | Abnomral Liver enzymes |
|  | Patient cannot have an active or recent (within 30 days) C difficile infection. | Active C Diff Infection |
|  | Patient must be over 18 years of age. | Age |
|  | Patient cannot have anemia (Hb < 9). | Anemia |
|  | Patient cannot have cardiovascular disease (MACE, stroke, CHF, arrythmia). | Cardiovascular Disease |
|  | Patient cannot be chronically taking NSAIDs. | chronic NSAIDS use |
|  | Patient cannot have history of colon dysplasia or cancer. | Colon Dysplasia |
|  | Patient cannot have history of colon dysplasia or cancer. | Colon Resection |
|  | Patient cannot be taking more than 30 mg of prednisone. | Current Use of Excluded Medications |
|  | Patient cannot be currently taking aTNF. | Current Use of Excluded Medications |
|  | Patient cannot be currently taking VDZ. | Current Use of Excluded Medications |
|  | Patient cannot be taking non-IBD immunosuppression medication including transplant medication (e.g. anakinra, abetacept, tacrolimus, cyclosporine, mycophenolate) . | Current Use of Excluded Medications |
|  | Patient cannot have diabetes 1 or poorly controlled diabetes 2 (A1c elevated, end organ complications) or macular edema. | Diabetes 1 or Poorly Controlled 2 |
|  | Patient cannot have disease extent limited to proctitis (involvement up to 15 cm). | Disease Extent |
|  | Patient cannot have history of thromboembolic event (arterial or venous). | History Of Thromboembolic Event |
|  | Patient cannot have history of rheumatologic disease (including lupus). | History of Rheumatologic Disease |
|  | Patient cannot have history of infection (zoster, hepatitis, HIV, TB, or serious infection). | Infection History |
|  | Patient cannot have Leukopenia (WBC < 3.5). | Leukopenia |
|  | Patient cannot have malignancy history (solid or liquid). | Malignancy History |
|  | Patient cannot have Nontuberculuous mycobacterial infection or serious opportunistic infection. | Nontuberculous Mycobacterial Infection or Serious Opportunistic Infection |
|  | Patient cannot have renal insufficiency (GFR > 30). | Renal Insuffiency |
|  | Patient cannot be starting or be on a not stable dose of Thiopurine/ MTX. | Thiopurine/ MTX Use |
|  | Patient cannot be taking topical/ rectal 5-ASA (Canasa, Rowasa). | Topical/ Rectal 5-ASA |
|  | Patient cannot be taking topical/ rectal steroids (budesonida foam, hydrocort enema). | Topical/ Rectal Steroids |
| **True North** | Patient cannot have an abnormal AST/ ALT (>2x ULN). | Abnomral Liver enzymes |
|  | Patient cannot have an active or recent (within 30 days) C difficile infection. | Active C diff Infection |
|  | Patient cannot be 75 years of age or over. | Age |
|  | Patient cannot have anemia (Hb < 9). | Anemia |
|  | Patient cannot have cardiovascular disease (MACE, stroke, CHF, arrythmia). | Cardiovascular Disease |
|  | Patient cannot be chronically taking NSAIDs. | chronic NSAIDS use |
|  | Patient cannot have history of colon dysplasia or cancer. | Colon Dysplasia |
|  | Patient cannot have had a colon resection or ostomy. | Colon Resection |
|  | Patient cannot be taking more than 30 mg of prednisone. | Current Use of Excluded Medications |
|  | Patient cannot be starting or be on a not stable dose of Thiopurine/ MTX. | Current Use of Excluded Medications |
|  | Patient cannot be currently taking aTNF. | Current Use of Excluded Medications |
|  | Patient cannot be currently taking VDZ. | Current Use of Excluded Medications |
|  | Patient cannot be currently taking a Jak inhibitor (tofa, upa). | Current Use of Excluded Medications |
|  | Patient cannot be currently taking an S1P (ozanimod, etrasimod). | Current Use of Excluded Medications |
|  | Patient cannot be taking topical/ rectal steroids (budesonida foam, hydrocort enema). | Current Use of Excluded Medications |
|  | Patient cannot be taking topical/ rectal 5-ASA (Canasa, Rowasa). | Current Use of Excluded Medications |
|  | Patient cannot be taking class 1A or class III anti arrthymic drugs. | Current Use of Excluded Medications |
|  | Patient cannot have diabetes 1 or poorly controlled diabetes 2 (A1c elevated, end organ complications) or macular edema. | Diabetes 1 or Poorly Controlled 2 |
|  | Patient cannot have disease extent limited to proctitis (involvement up to 15 cm). | Disease Extent |
|  | Patient cannot have history of thromboembolic event (arterial or venous). | History Of Thromboembolic Event |
|  | Patient cannot have history of rheumatologic disease (including lupus). | History of Rheumatologic Disease |
|  | Patient cannot have history of infection (zoster, hepatitis, HIV, TB, or serious infection). | Infection History |
|  | Patient cannot have Leukopenia (WBC < 3.5). | Leukopenia |
|  | Patient cannot have malignancy history (solid or liquid). | Malignancy History |
|  | Patient cannot have Nontuberculuous mycobacterial infection or serious opportunistic infection. | Nontuberculous Mycobacterial Infection or Serious Opportunistic Infection |
|  | Patient cannot be currently taking or have taken natalzumab. | Prior Use of Excluded Medication |
|  | Patient cannot have renal insufficiency (GFR > 30). | Renal Insuffiency |
| **U. Accomplish & U. Achieve** | Patient cannot have an abnormal AST/ ALT (>2x ULN). | Abnomral Liver enzymes |
|  | Patient cannot be 75 years of age or over. | Age |
|  | Patient cannot have anemia (Hb < 9). | Anemia |
|  | Patient cannot have an active or recent (within 30 days) C difficile infection. | C Difficile Infection |
|  | Patient cannot have cardiovascular disease (MACE, stroke, CHF, arrythmia). | Cardiovascular Disease |
|  | Patient cannot have history of colon dysplasia or cancer. | Colon Dysplasia |
|  | Patient cannot have had a colon resection or ostomy. | Colon Resection |
|  | Patient cannot be taking more than 30 mg of prednisone. | Current Use of Excluded Medications |
|  | Patient cannot be currently taking aTNF. | Current Use of Excluded Medications |
|  | Patient cannot be currently taking VDZ. | Current Use of Excluded Medications |
|  | Patient cannot be currently taking UST/ RISA. | Current Use of Excluded Medications |
|  | Patient cannot be taking non-IBD immunosuppression medication including transplant medication (e.g. anakinra, abetacept, tacrolimus, cyclosporine, mycophenolate) . | Current Use of Excluded Medications |
|  | Patient cannot have diabetes 1 or poorly controlled diabetes 2 (A1c elevated, end organ complications) or macular edema. | Diabetes 1 or Poorly Controlled 2 |
|  | Patient cannot have disease extent limited to proctitis (involvement up to 15 cm). | Disease Extent |
|  | Patient cannot have history of thromboembolic event (arterial or venous). | History Of Thromboembolic Event |
|  | Patient cannot have history of rheumatologic disease (including lupus). | History of Rheumatologic Disease |
|  | Patient cannot have history of infection (zoster, hepatitis, HIV, TB, or serious infection). | Infection History |
|  | Patient cannot have Leukopenia (WBC < 3.5). | Leukopenia |
|  | Patient cannot have malignancy history (solid or liquid). | Malignancy History |
|  | Patient cannot have Nontuberculuous mycobacterial infection or serious opportunistic infection. | Nontuberculous Mycobacterial Infection or Serious Opportunistic Infection |
|  | Patient cannot be chronically taking NSAIDs. | NSAIDs |
|  | Patient cannot be taking or have taken a Jak inhibitor. | Prior Use of Excluded Medication |
|  | Patient cannot have renal insufficiency (GFR > 30). | Renal Insuffiency |
| **Unifi** | Patient cannot have an abnormal AST/ ALT (>2x ULN). | Abnomral Liver enzymes |
|  | Patient cannot have an active or recent (within 30 days) C difficile infection. | Active C diff Infection |
|  | Patient must be over 18 years of age. | Age |
|  | Patient cannot have anemia (Hb < 9). | Anemia |
|  | Patient cannot have cardiovascular disease (MACE, stroke, CHF, arrythmia). | Cardiovascular Disease |
|  | Patient cannot be chronically taking NSAIDs. | Chronic NSAIDS use |
|  | Patient cannot have history of colon dysplasia or cancer. | Colon Dysplasia |
|  | Patient cannot have had a colon resection or ostomy. | Colon Resection |
|  | Patient cannot be taking more than 30 mg of prednisone. | Current Use of Excluded Medications |
|  | Patient cannot be currently taking aTNF. | Current Use of Excluded Medications |
|  | Patient cannot be currently taking VDZ. | Current Use of Excluded Medications |
|  | Patient cannot have diabetes 1 or poorly controlled diabetes 2 (A1c elevated, end organ complications) or macular edema. | Diabetes 1 or Poorly Controlled 2 |
|  | Patient cannot have disease extent limited to proctitis (involvement up to 15 cm). | Disease Extent |
|  | Patient cannot have history of thromboembolic event (arterial or venous). | History Of Thromboembolic Event |
|  | Patient cannot have history of rheumatologic disease (including lupus). | History of Rheumatologic Disease |
|  | Patient cannot have history of infection (zoster, hepatitis, HIV, TB, or serious infection). | Infection History |
|  | Patient cannot have Leukopenia (WBC < 3.5). | Leukopenia |
|  | Patient cannot have malignancy history (solid or liquid). | Malignancy History |
|  | Patient cannot have Nontuberculuous mycobacterial infection or serious opportunistic infection. | Nontuberculous Mycobacterial Infection or Serious Opportunistic Infection |
|  | Patient cannot be taking or have taken UST/ RISA. | Prior Use of Excluded Medication |
|  | Patient cannot have renal insufficiency (GFR > 30). | Renal Insuffiency |
| **Varsity** | Patient cannot have an abnormal AST/ ALT (>2x ULN). | Abnomral Liver enzymes |
|  | Patient cannot have an active or recent (within 30 days) C difficile infection. | Active C diff Infection |
|  | Patient must be under 85 years of age. | Age |
|  | Patient cannot have anemia (Hb < 9). | Anemia |
|  | Patient cannot have cardiovascular disease (MACE, stroke, CHF, arrythmia). | Cardiovascular Disease |
|  | Patient cannot be chronically taking NSAIDs. | Chronic NSAIDs use |
|  | Patient cannot have history of colon dysplasia or cancer. | Colon Dysplasia |
|  | Patient cannot have had a colon resection or ostomy. | Colon Resection |
|  | Patient cannot be taking more than 30 mg of prednisone. | Current Use of Excluded Medications |
|  | Patient cannot be taking non-IBD immunosuppression medication including transplant medication (e.g. anakinra, abetacept, tacrolimus, cyclosporine, mycophenolate) . | Current Use of Excluded Medications |
|  | Patient cannot be taking topical/ rectal steroids (budesonida foam, hydrocort enema). | Current Use of Excluded Medications |
|  | Patient cannot be taking topical/ rectal 5-ASA (Canasa, Rowasa). | Current Use of Excluded Medications |
|  | Patient cannot have diabetes 1 or poorly controlled diabetes 2 (A1c elevated, end organ complications) or macular edema. | Diabetes 1 or Poorly Controlled 2 |
|  | Patient cannot have disease extent limited to proctitis (involvement up to 15 cm). | Disease Extent |
|  | Patient cannot have history of thromboembolic event (arterial or venous). | History Of Thromboembolic Event |
|  | Patient cannot have history of rheumatologic disease (including lupus). | History of Rheumatologic Disease |
|  | Patient cannot have history of infection (zoster, hepatitis, HIV, TB, or serious infection). | Infection History |
|  | Patient cannot have Leukopenia (WBC < 3.5). | Leukopenia |
|  | Patient cannot have malignancy history (solid or liquid). | Malignancy History |
|  | Patient cannot have Nontuberculuous mycobacterial infection or serious opportunistic infection. | Nontuberculous Mycobacterial Infection or Serious Opportunistic Infection |
|  | Patient cannot be taking or have taken Vedolizumab. | Prior Use of Excluded Medication |
|  | Patient cannot be taking or have taken Adalimumab. | Prior Use of Excluded Medication |
|  | Patient cannot have renal insufficiency (GFR > 30). | Renal Insuffiency |

Supplemental Table 3: Key exclusion criteria from the TRUE NORTH³, U-ACCOMPLISH & U-ACHIEVE⁴, OCTAVE⁵, ELEVATE⁶, UNIFI⁷, VARSITY⁸, and LUCENT clinical trials, and their corresponding definitions as applied in this study.
